# Being paid relatively well most of the time: Negatively skewed payments are more satisfying

**DOI:** 10.3758/s13421-016-0604-0

**Published:** 2016-03-31

**Authors:** James Tripp, Gordon D. A. Brown

**Affiliations:** Department of Psychology, University of Warwick, Coventry, UK

**Keywords:** Satisfaction, Payment, Skewness, Range frequency, Reference point

## Abstract

How does the structure of a series of payments influence its recipient’s satisfaction? A common hypothesis is that each payment will be compared with a single “standard” or “reference” payment (e.g., the average payment). Cognitive models of judgment such as range frequency theory predict in contrast that the entire payment distribution will influence evaluation of each individual payment. Two experiments examined satisfaction with a series of payments. In both experiments, most payments were either relatively high in the experienced distribution (the distribution was negatively skewed) or relatively low (positively skewed). The total and average payment was held constant. Experiment 1 found that average satisfaction with individual payments was higher when the payments were negatively skewed, consistent with range frequency theory, and earlier findings were extended by comparing range frequency theory with a range-based model, a rank-based model, and a reference point model at the individual level. Experiment 2 examined satisfaction with whole sequences of payments and found that receiving a negatively skewed sequence was more satisfying overall than receiving a positively skewed sequence. It is concluded that negatively skewed payment distributions are more satisfying, as predicted by cognitive models of judgment.

What effect does the structure of a sequence of payments have on satisfaction with those payments? Consider a case where a person receives regular payments for work punctuated by occasional larger amounts (e.g., bonuses). Under such a system the majority of payments received are at the lower end of the range of experienced payments—that is, the payment distribution is positively skewed. However, recipients’ overall evaluations of an experienced sequence of payments may be adversely affected under such conditions. Some cognitive models of context-based judgment, such as range frequency theory, suggest that occasional high payments may overshadow the more frequent lower payments. Intuitively, it may be dissatisfying to receive, on the majority of occasions, payments that are at the lower end of the range of payments ever received (Parducci, 1968). If the overall amount that is received is held constant, would people be more satisfied with a negatively skewed distribution of payments, in which relatively high payments occur most of the time, even if the overall amount of pay was the same, as Parducci suggested?

Here we report two experiments that test the predictions of cognitive models of context-based judgment for satisfaction with different payment structures. In both experiments participants receive either a negatively or a positively skewed series of payments and reported their satisfaction with individual payments they receive (Experiment 1) or with the sequence of payments as a whole (Experiments 1 and 2).

The first experiment replicates and extends work by Parducci (1968), who found that, consistent with range frequency theory, the average satisfaction with individual payments from a sequence is greater when the sequence is negatively skewed than when it is positively skewed. We go beyond Parducci in using likelihood-based model fitting at the level of individual participants. This allows model-based analysis of individual differences to examine whether existing results based on mean data might reflect the use of different strategies by different participants. The model-based analysis also allows evaluation of the alternative predictions of single reference point models and pure rank-based models. We also extend Parducci (1968) in examining both satisfaction with individual payments and satisfaction with whole sequences of payments. We replicate Parducci’s finding that the average satisfaction with individual payments is higher when the payments are drawn from a negatively skewed distribution but fail to find an effect of skew on summary evaluations of the whole sequence of payments. We also find that the individual data from most participants are best fit by range frequency theory but that a substantial minority of participants produced data that were best fit by either a range-only or a rank-only model.

Experiment 2 is motivated by a body of research suggesting that online evaluations and retrospective evaluations may involve different psychological processes (e.g., Hastie & Park, 1986). We examined whether summary evaluations of the whole sequence of payments would be affected by skew when, in contrast to Experiment 1, satisfaction with individual payments was not elicited. We gave participants monetary payments rather than the tokens used in Experiment 1 and asked participants to recall the payment structure they received to determine whether skewness information was accessible when making summary evaluations. In Experiment 2, negatively skewed distributions were more positively evaluated.

## Previous findings

### Within-individual comparisons

Few studies have directly examined satisfaction with skewed payment structures. A seminal paper by Parducci (1968) gave participants either positively or negatively skewed payment structures. On each of many trials, participants received a payment after selecting one of three cards. After each payment the participants rated their satisfaction with that payment. The average of the satisfaction ratings was higher when the payment structure was negatively skewed. This finding is important because ratings along hedonic scales such as satisfaction or well-being is sometimes interpreted as an important component of utility (Oswald & Wu, 2010; Sandvik, Diener, & Seidlitz, 1993). However, the study did not examine participants’ satisfaction with the overall sequence of payments that they received (e.g., by asking them for a summary satisfaction judgment at the end of the study). The study also did not examine individual differences (i.e., to discover whether all participants’ response patterns were individually best described by range frequency theory) and does not allow us to determine whether the range-only model, or the range frequency theory compromise, best fit the data.

Smith, Diener and Wedell (1989) found that summary evaluations were higher for negatively skewed payment structures. Smith et al. asked participants to rate the happiness they would experience with a series of six hypothetical tips and then to rate their overall happiness with the distribution of tips. The tip amounts were either positively or negatively skewed. The mean of the payments ratings and the summary judgment were both higher when the distributions of tips were negatively skewed. These results, like those of Parducci (1968; see also Parducci, 1995) are consistent with the expectation that negative skewed payment distributions will be preferred but (unlike Parducci, 1968) used only hypothetical payments that were not experienced sequentially.

### Across-individual comparisons

A large literature has examined the effects of income distribution (inequality) on various measures of well-being (e.g., Wilkinson & Pickett, 2010). Given that real-world income distributions are almost invariably positively skewed, research that has examined the effects of income inequality on happiness (e.g., Alesina, Di Tella, & MacCulloch, 2004; Hagerty, 2000) is relevant to across-individual comparisons and to preferences for greater or lesser amounts of positive skew in an income distribution. It is, however, of limited relevance to the current study even though the payment of others can influence judgments of one’s own payments (for a review, see Rynes & Gerhart, 2003).

### Contextual models of judgment

A central assumption underpinning the present research is that the context (here, the overall payment distribution) within which a payment is received will influence the satisfaction judgment associated with its receipt. Within cognitive psychology, there are several different models of how judgments are made within a context (e.g., Vlaev, Chater, Stewart, & Brown, 2011). Here we briefly review the most relevant models of contextual judgment and describe their predictions for the evaluation of different payment structures.

#### Reference-level model

One possibility is that each payment is compared to some average, “typical,” or “reference-level” amount. The idea that subjective judgments of payments involve comparison of each to a single reference point can be seen as an application of the adaptation level model (Helson, 1964) and is shown in Eq. 1:

1 $$ {J}_i=k\cdot \left({S}_i-\overline{S}\right), $$

where the judgment *J*_*i*_ of a stimulus *S*_*i*_ depends on its distance from the “adaptation level,” which will in turn depend on the average payment $\overline{S}$ and perhaps also on other background or remembered stimuli. A constant *k* scales responses to fit within the range of possible responses. Within the income satisfaction literature several studies have suggested that satisfaction with one’s income depends on its relation to an average or “reference” income (e.g., Clark & Oswald, 1996; Luttmer, 2004), but these studies relate to across-individual comparisons rather than the within-individual comparisons that form the focus of the present paper.

The predictions of the reference-level model for the evaluation of positively and negatively skewed payment distributions depend on the reference payment (or adaptation level) to which all payments are compared. If the reference payment is the mean payment, as is typically assumed, then the model predicts equal satisfaction for sequences with the same mean (Brickman & Campbell, 1971).

#### Relative-rank model

Alternatively, people may evaluate individual payments according to their relative rank within the distribution of expected or experienced payments. This approach is consistent with the “decision by sampling” model (Stewart, Chater, & Brown, 2006), according to which economic quantities such as payments are evaluated by counting up the number of higher and lower quantities that are present in a mental comparison sample. Several studies support a relative-rank account of judgments of wages (Brown, Gardner, Oswald, & Qian, 2008) and life satisfaction (Boyce, Brown, & Moore, 2010), finding that people gain satisfaction from an income to the extent that it ranks higher than others rather than (or as well as) from its absolute amount. However, such accounts, like the reference-level model, have not generally been applied to within-individual judgments of payment sequences.

The relative rank *F*_*i*_ of the *i*th largest payment out of *n* is given by:

2 $$ {F}_i=\frac{i-1}{N-1}. $$

Thus, applied to reward evaluation, the predicted satisfaction with a given payment will be the number of lower payments divided by the total number of payments. The relative-rank model predicts no difference in overall satisfaction if the number of items in the series is the same—distributional changes leave rank information unchanged. Consequently, a pure relative-rank model predicts no effect of the skewness of the payment structure (although, see Brown & Matthews, 2011; Brown, Wood, Ogden, & Maltby, 2015, for accounts of how apparent range effects may emerge from rank-based processes).

#### Range model

The third type of model assumes that the position of a payment within the range of experienced or expected payments will influence satisfaction with that payment. Changing the skew of a payment structure alters the range position of the individual payments. In a positively skewed structure, most payments are in the lower portion of the range, while in a negatively skewed payment structure, most of the payments are in the upper portion of the range. Consequently, evaluations based on the range position of the payments will be higher (on average) in a negatively skewed payment structure (Parducci & Wedell, 1986). A range-based prediction is given by:

3 $$ {R}_i=\frac{S_i-{S}_{\min }}{S_{\max }-{S}_{\min }}, $$

where *R*_*i*_ is the range-based judgment of stimulus *S*_*i*_, given the smallest (*S*_min_) and largest (*S*_max_) stimulus.

The range-based prediction for satisfaction with an individual payment thus depends on the distance of a payment from the smallest payment divided by the total range of payments. Range effects have been reported in the salary literature (Highhouse, Luong, & Sarkar-Barney, 1999; Rynes, Sara, & Heneman, 1983).

#### Range frequency theory

The range-frequency model (e.g., Parducci, 1965) combines rank- and range-based predictions. Judgments predicted by the range-frequency model are a weighted compromise between range-based and rank-based responses, as shown in the equation below:

4 $$ {J}_i=w\cdot {R}_i+\left(1-w\right)\cdot {F}_i, $$

where *R*_*i*_ and *F*_*i*_ are as in Eqs. 2 and 3.

The relative weighting of range and rank is specified by the *w* parameter. When *w* equals 1 predictions are as for the range model, and when *w* equals 0 predictions are as for the relative-rank model. Predictions based solely on relative rank (*w =* 0) are the same for differently skewed payment structures. However, predictions based on the range position of stimuli will be for higher average satisfaction in a negatively skewed payment structure because there are more payments in the upper portion of the range in a negatively skewed compared to a positively skewed structure. The range-frequency model (as applied to payment evaluation) predicts higher average satisfaction in the negatively skewed payment structure as the weighting parameter approaches 1.

There is considerable support in the wider judgment literature for the range-frequency approach. Studies examining judgments of drink sweetness (Riskey, Parducci, & Beauchamp, 1979), hypothetical tips (Wedell & Parducci, 1988), payments (Parducci, 1968, 1995) and wages (Brown et al., 2008), personalities (Wood, Brown, Maltby, & Watkinson, 2012), and many other types of stimuli (Birnbaum, Parducci, & Gifford, 1971; Parducci, Calfee, Marshall, & Davidson, 1960; Wedell & Parducci, 1988; Wedell, Parducci, & Geiselman, 1987) show response patterns that are predicted by the range-frequency model but are inconsistent with either rank-only or range-only models.

The models described above make different predictions for evaluations of skewed payment structures. The mean comparison and pure rank-based models predict no effect of skewness unless additional assumptions are made. The range-only and range-frequency models (with *w* > 0) predict greater satisfaction with negatively skewed payment structures. These model predictions will be compared in Experiment 1. Moreover, previous research has not examined individual differences in strategies: a good fit of range frequency theory to data that have been averaged across participants might mask a pattern in which some participants adopt a range-only strategy while others adopt a rank-only strategy.^1^ Experiment 1 addresses this issue directly by undertaking model comparison at the level of the individual.

## Experiment 1

Following Parducci (1968), we asked participants to rate their satisfaction with each of a series of credit payments drawn from either a positively or negatively skewed payment structure. We extend Parducci (1968) by asking participants to rate their satisfaction with the outcome of the overall sequence of payments, not just their satisfaction with individual payments.

### Method

#### Participants

Forty University of Warwick undergraduates were split into two groups of 20 and paid five candies.

#### Procedure

Participants were seated at a table with three facedown cards placed on it. Below the cards was a satisfaction scale ranging from 1 (*very dissatisfied*) to 7 (*very satisfied*). Above the cards were three piles of candies with labels of 0–599 (four candies), 600–1,199 (five candies), and 1,200+ (six candies). Participants were told these labels were the number of credits required to win the corresponding pile of candies.

**Fig. 1:**
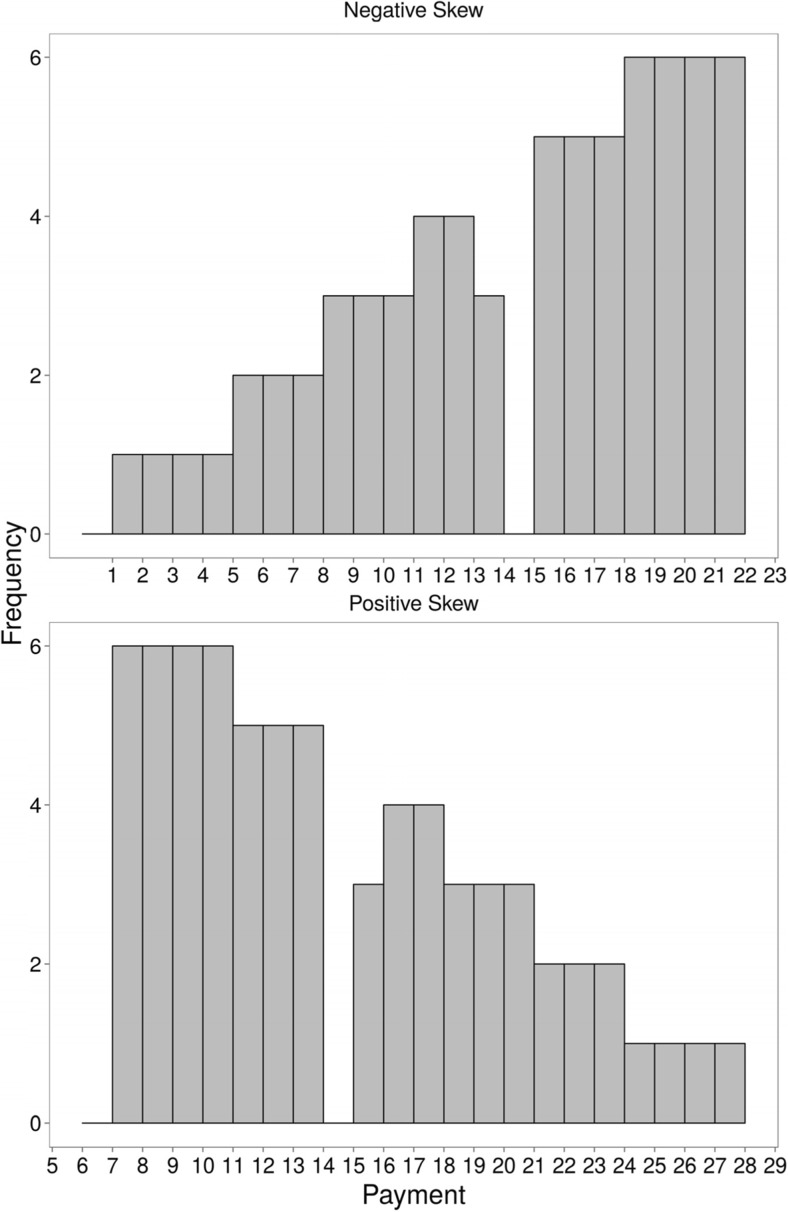
Payment distributions offered to participants in Experiments 1 and 2

Participants were separated into two groups: the positive skew and negative skew groups. The positive skew group were shown the top histogram in Fig. 1 and the negative skew group were shown the bottom histogram. Both groups were told—correctly—that they would receive the payment distribution depicted on the histogram; the histogram was then removed. To illustrate the procedure, each participant was first asked to choose one of the cards—all the cards had the value of 14 (the mean of the distribution), unbeknownst to the participant—turn it over and tell the experimenter their satisfaction with that number of credits. The order of the payments was randomly chosen, and decks were preshuffled. Then the cards were dealt from the top of the deck in groups of three and placed face down on the table.

The procedure of turning over a card and telling the experimenter his or her satisfaction with the associated reward was then repeated for each value in the payment distribution—69 times in total. A -cm × 1-cm paper token representing credit payment was given to the participant after turning over a card, and three new cards were dealt by the experimenter each time. After all the payments, the participant was (correctly) told that he or she had won 966 credits in total. The participant gave the experimenter all of the credit tokens that he or she had received in exchange for five candies, and the experimenter then asked the participant to rate his or her satisfaction with the overall amount of candies received.

### Results and discussion

The data of three participants were removed from the analysis due to a low correlation between the satisfaction rating given to credit payments and the value of the credit payments. The Spearman correlation coefficients between payments and satisfaction ratings for these participants were all less than .6, suggesting that these participants had misunderstood the task.

The mean trial-by-trial and the overall satisfaction ratings were entered into *t* tests. The average trial-by-trial satisfaction ratings were significantly higher for participants who received a negatively skewed credit distribution (*M* = 4.49, *SD* = 0.31) than for those who received a positively skewed credit distribution (*M* = 3.77, *SD* = 0.60), *F*(1, 35) = 21.76, *p* < .001, η^2^ = .38 . This difference replicates the finding of Parducci (1968). However, there was no significant difference between the ratings of satisfaction with the overall reward received given at the end of the experiment by participants in the positive (*M* = 4.93, *SD* = 0.81) and negative skew (*M* = 4.91, *SD* = 0.85) conditions, *F*(1, 35) = 0.17, *p* = .69.

#### Model comparison

Model fitting was undertaken to determine whether range frequency theory fit the satisfaction ratings for credit payments better than did competing models. We answer this question by (a) comparing the range= frequency model to rank-only and range-only models and (b) comparing the range=frequency model to the reference-level model.

The model comparison used a maximum likelihood method. Each participant’s response is assumed to be the same as a model’s prediction plus normally distributed noise. The parameters of the model (i.e., the *w* parameter in the range-frequency model) and the standard deviation of the normally distributed noise were allowed to vary freely. To compare the models, we used the Bayesian information criterion (BIC) to penalize the maximum likelihood using the number of parameters, *k*, and observations, *n*,

5 $$ BIC=-2 \ln L+k\bullet \ln (n). $$

We interpret these BIC values using the “weight of evidence” metric. The metric is the ratio of the difference in BIC value between a model and the best model, BIC *Δ*_*i*_, to the summed difference between all models and the best model,

6 $$ BIC{w}_i=\frac{e^{\left(-0.5\cdot BIC{\varDelta}_i\right)}}{{\displaystyle {\sum}_{r=1}^R}{e}^{\left(-0.5\cdot BIC{\varDelta}_r\right)}}, $$

where values closer to 1 correspond to more evidence in favor of a model relative to the other candidate models.

We varied the weighting parameter in the range-frequency model to compare the rank only (*w* = 0) and range only (*w* = 1) models to the range-frequency model (0 ≤ *w* ≤ 1). Predictions for the reference-level model assumed the reference level was 14—the mean of the payment distribution.

**Fig. 2:**
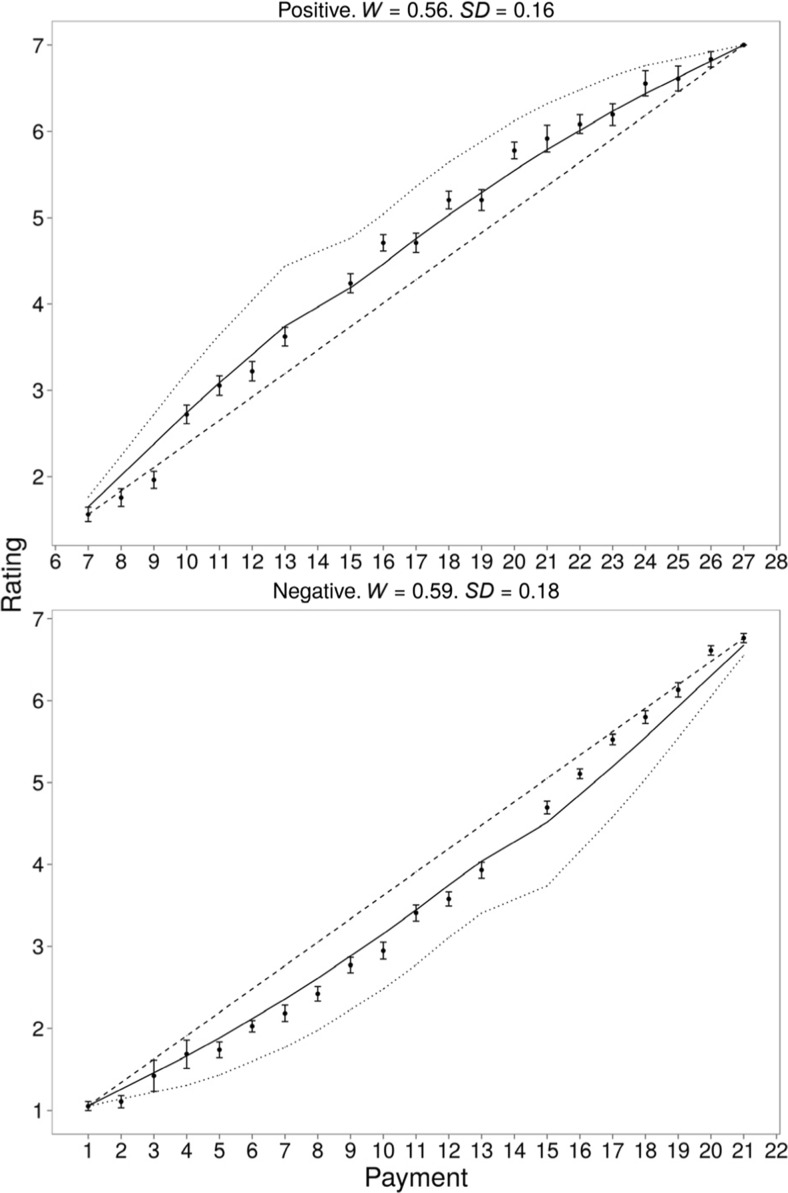
Mean responses to payments in Experiment 1 and predictions of the range-frequency (solid line), range-only (dashed line) and rank-only (dotted line) models

At the group level, data from the positive and negative skew conditions were fit separately. Figure 2 shows the predictions of each model against the mean data. For the positive skew data, the range-frequency model (-2lnL = -17.31, BIC = -8.84, BICw =1) outperformed both the range-only (-2lnL = 20.55, BIC = 24.78, BICw = 0) and the rank-only (-2lnL = 28.93, BIC = 33.16, BICw = 0) models. For the negative skew data, the range-frequency model (-2lnL = -12.40, BIC = -3.93, BICw = 1) again outperformed both the range-only (-2lnL = 20.25, BIC = 24.48, BICw = 0) and rank-only (-2lnL = 33.42, BIC = 37.65, BICw = 0) models.

At the individual level we calculated the BIC of each model for every participant. The BIC values were used for two comparisons. First, we considered the range-frequency, range-only, and rank-only models. The BIC weight for the range-frequency model was highest for most participants (range-frequency model: 21 participants, range only: 8 participants, rank only: 8 participants). In our second comparison, the BIC weights from the range-frequency model were the highest (range-frequency model: 21 participants, reference level: 16 participants). Our individual-level analysis suggests that the majority of participants combine the range and rank position of items as predicted by range frequency theory.

### Discussion

The finding that the average satisfaction with individual payments was higher when payments came from a negatively skewed distribution replicates Parducci (1968) and is consistent with the predictions of range frequency theory. Model-fitting demonstrated that the range-frequency model accounted for the data better than did reference-level, range-only, or rank-only models at both the group and individual level.

However, the main finding, that participants preferred a negatively skewed payment distribution, must be qualified in two important ways. First, individual-level analysis found that 21 participants were best fit by range frequency theory, with another eight being best fit by a range-only model. Thus, the remaining eight of the participants—those whose data were best fit by a rank-only model—would be predicted to be unaffected by the skew of the payment distribution they received.

Second, participants’ stated satisfaction with the distribution as a whole was not affected by skew. While the absence of a skew effect is inconsistent with literature reviewed above and with evidence of averaging in summary hedonic evaluations (Miron-Shatz, 2009), we hypothesize that the use of online responses may have distorted any effects of the skewness of the distribution. Online ratings in some studies have reduced or removed the effect of stimulus structure (Ariely, 1998; Ariely & Carmon, 2000; Ariely & Zauberman, 2000, 2003). Moreover, a considerable body of research, originating with Hastie and Park (1986), has emphasized the difference between online and retrospective evaluations (see, e.g., Aldrovandi, Poirier, Kusev, & Ayton, 2015; Montgomery & Unnava, 2009). Furthermore, participants may (having already stated their satisfaction with the individual payments they received) interpreted the task demands such that they produced an evaluation of different aspects of the experiment in their summary evaluation. For example, all participants received five candies as their earnings-related reward at the end of the experiment, where the other possible rewards were four or six candies. They may therefore have been expressing their satisfaction with receiving the middle one of the three possible rewards.

We address these possibilities directly in the next experiment by asking participants only for retrospective judgments of their satisfaction with the entire summed sequence of monetary payments they receive.

## Experiment 2

In Experiment 2, the online ratings used in Experiment 1 were replaced by a task in which the participants merely entered the credit payment they received on each trial. This was done to ensure attention to the individual rewards while removing the requirement to evaluate individual payments at the time they are received. As before, participants provided a summary evaluation of their satisfaction with the overall distribution of payments they received. We predict that the participants would be more satisfied with the overall outcome of a negatively skewed payment distribution. We also asked participants to recall the payments they received to verify that the skew of the payment distribution was accessible in memory when overall ratings were made. Moreover, as a robustness check, we examined whether the predicted preference for negatively skewed distributions would remain when dissatisfaction, rather than satisfaction, judgments were elicited.

### Method

#### Participants

The sample consisted of 84 undergraduates from the University of Warwick who were paid £4.16 and separated into four groups of 21 (the skewness of the payment distribution was either positive or negative; the Likert scale used by participants asked for either satisfaction or dissatisfaction). Position of the recall task in the procedure (before or after provision of an overall satisfaction rating) was counterbalanced.^2^

#### Design and procedure

The experiment was presented in a web browser. Participants were shown histograms depicting the skewed payment structures; these were identical to those used in Experiment 1 except that values were all half as large. Three cards were shown onscreen, whereupon the participant clicked on a card and then typed in the value of card in a box displayed below. The process of clicking on a card and typing in the amount displayed on the card was repeated for every payment in either the negatively or positively skewed distribution. Participants then recalled the payments they saw on a piece of papers and rated their overall satisfaction on a Likert scale. The Likert scales ran from either 1 (*very satisfied*) to 7 (*very dissatisfied*) or 1 (*very dissatisfied*) to 7 (*very satisfied*).

### Results and discussion

To examine the influence of skewness on overall ratings we ran 2 (skew) × 2 (Likert scale) ANOVA. There was a significant main effect of skew, *F*(1, 80) = 13.53, *p* < .001, η^2^ = .14, with participants being more satisfied after receiving a negatively skewed payment distribution (*M* = 5.31, *SD* = 1.22) compared to a positively skewed payment distribution (*M* = 4.29, *SD* = 1.29). The Likert scale had no significant main effect, *F*(1, 80) = 0.001, *p* = .97, nor was there a significant interaction, *F*(1, 80) = 0.23, *p* = .63. Thus, skewness did influence overall satisfaction when participants received monetary payments and did not rate the individual payments.

We then calculated the skewness of the payments recalled by participants using the following formula,^3^

7 $$ \frac{{\displaystyle \sum }{\left(x - \overline{x}\right)}^3/n}{{\left(\raisebox{1ex}{${\displaystyle \sum }{\left(x - \overline{x}\right)}^2$}\!\left/ \!\raisebox{-1ex}{$n$}\right.\right)}^{\frac{3}{2}}}, $$

where *x* is a recalled payment, $\overline{x}$ is the mean of the recalled payments and *n* is the number of recalled payments. An examination of the recall data revealed that all participants recalled at least five payments (*M* = 29.14, *SD* = 15.94), and only one participant failed to recall multiple instances of at least one payment (average number of times each payment was recalled across participants = 2.23, *SD* = 1.86). The skewness of the distributions participants recalled matched the skewness of the payment distribution they received in both the positive (*M* = 0.46, *SD* = 0.32) and negative skew (*M* = -0.70, *SD* = 0.40) condition.

In summary, Experiment 2 found that participants were more satisfied overall when receiving a negatively skewed payment distribution.

## General discussion

Two experiments showed that negatively skewed payment distributions are judged to be more satisfying. This effect holds when the average satisfaction with individual payments is examined (Experiment 1; Parducci, 1968) and is estimated to hold for about 90% of participants based on individual-level model fitting (Experiment 1). It is also seen when a whole series of payments must be given a summary evaluation (Experiment 2) and applies when dissatisfaction rather than satisfaction judgments are elicited (Experiment 2).

At a theoretical level, the findings challenge reference-level models according to which payments are judged relative to some mean or reference-level payment. Instead, the results appear consistent with an interpretation in terms of range frequency theory (e.g., Parducci, 1968, 1995).

We note that the preference for negatively skewed distributions of rewards contrasts with skew preferences for lotteries. Both humans (Burke & Tobler, 2011; Golec & Tamarkin, 1998; Symmonds, Wright, Bach, & Dolan, 2011) and animals (Caraco & Chasin, 1984; Coombs & Pruitt, 1960) prefer lotteries with positively skewed outcomes when the mean expected gain is held constant. This contrast might appear surprising, if a judgment of satisfaction with a payment distribution is akin to judgment of a range of risky outcomes (i.e., a lottery), with the different payments playing the role of different outcomes and the relative frequency of a given payment being akin to its probability. Responses to payment structures may, however, be quite different to preferences for lotteries, for at least two reasons. First, the judgment of risky prospects, where the outcome is not under the control of the person making a judgment or choice, seems quite different from a case where payments are earned and are assumed to reflect effort. Second, several cognitive biases (such as loss aversion) characterize anticipated feelings prior to an outcome rather than the reactions that are actually experienced following an outcome or outcomes (Gilbert, Morewedge, Risen, & Wilson, 2004; Kermer, Driver-Linn, Wilson, & Gilbert, 2006). People are subject to “affective forecasting errors” such that, for example, they overestimate the intensity of the negative feelings they will experience when they suffer a loss. Moreover, described and experienced outcomes are often differently evaluated (Hertwig, Barron, Weber, & Erev, 2004). Therefore, people’s preferences for positively and negatively skewed outcomes in described lotteries may not relate to their satisfaction with experienced distributions of probabilistic payments.

At a practical level, the findings suggest that payment satisfaction can be altered by manipulating only the skew of the payment structure. Although our laboratory experiment differs greatly from real-world reward structures, the results may have implications for the relation between satisfaction and typical bonus-style schemes in which the highest payments are the least frequent. Existing schemes typically ignore the possible effects of distribution, which may moderate the effects of incentive schemes, although further studies would be needed to demonstrate this in a natural context.

## Footnotes

1 Simulations (not reported in detail here) confirmed that the mean of data generated by a mixture of range-only and rank-only simulated participants is best fit by range frequency theory.

2 This presentation order variable did not lead to a main effect or any interactions and hence is not discussed further.

3 The formula is taken from the skewness function in the “moments” R package. The summed deviance of the data from the mean is normalized by the sample size in both the numerator and denominator. When most of the data are smaller than the mean (as for a negatively skewed distribution) then the sign of the deviance is negative. Cubing this deviance in the numerator and squaring the deviance in the denominator makes the two unequal so that the result represents the skewness of the distribution. Raising the denominator to the power 3/2 constrains the ratio of the values so that they accurately represent skewness.
